# What do guidelines and systematic reviews tell us about the management of medically unexplained symptoms in primary care?

**DOI:** 10.3399/bjgpopen17X101061

**Published:** 2017-10-04

**Authors:** Tim C olde Hartman, Marianne Rosendal, Aase Aamland, Henriette E van der Horst, Judith GM Rosmalen, Chris D Burton, Peter LBJ Lucassen

**Affiliations:** 1 GP, Department of Primary and Community Care, Radboud University Nijmegen Medical Center, Nijmegen, The Netherlands; 2 GP, Research Unit of General Practice, Institute of Public Health, University of Southern Denmark, Odense, Denmark; 3 GP, Research Unit for General Practice, Uni Research Health, Bergen, Norway; 4 GP, Research Unit for General Practice, Uni Research Health, Bergen, Norway; 5 GP, Department of General Practice and Elderly Care Medicine, VU University Medical Centre, Amsterdam, Netherlands; 6 Professor of Psychosomatic Research, Department of Psychiatry and Internal Medicine, University of Groningen, University Medical Center Groningen, Groningen, Netherlands; 7 GP, Academic Unit of Primary Medical Care, University of Sheffield, Sheffield, UK; 8 GP, Department of Primary and Community Care, Radboud University Nimegen Medical Centre, Nijmegen, The Netherlands

**Keywords:** medically unexplained symptoms, primary care, doctor-patient relationship, doctor-patient communication, guidelines, review

## Introduction

Medically unexplained symptoms (MUS) are symptoms for which the origin remains unclear despite adequate history taking, physical examination, and additional investigations.^1^ An estimated 3–11% of patients visiting general practice repeatedly consult their GP for MUS.^2,3^ MUS exist along a continuum ranging from self-limiting symptoms, to recurrent and persistent symptoms, through to symptom disorders.^4^ Although there are various terms for the condition, for example unexplained physical symptoms, functional symptoms, or somatoform symptoms, we have chosen to use MUS in this article because this is the most frequently used term. This review aims to address current problems with the management of undifferentiated MUS; specific syndromes within the MUS spectrum, such as chronic fatigue syndrome and irritable bowel syndrome, are excluded from discussion.

Patients with persistent MUS suffer from their symptoms, are functionally impaired, and are at risk of potentially harmful additional testing and treatment.^5^ Furthermore, these patients commonly express dissatisfaction with the medical care they receive during their illness.^6^ They feel stigmatised and not taken seriously.^7^ GPs often experience patients with persistent MUS as difficult and frustrating to manage.^8^ In addition, MUS are associated with reduced health-related quality of life, higher healthcare and social costs, and costs associated with lost productivity.^9,10^

The effects of many treatment strategies have been studied in recent decades. However, not all interventions are acceptable or feasible in routine primary care. In the light of the central role of the GP in managing MUS, we will discuss the importance of consultation skills and the effects of specific treatments in primary care. We will do this by way of a narrative review using available national guidelines and Cochrane Reviews in this field.

### Clinical guidelines on MUS in primary care

In recent years several guidelines on MUS have been published: a Dutch multidisciplinary guideline (2010);^11^ a German multidisciplinary guideline (2012);^12^ Dutch^13^ and Danish^14^ general practice guidelines (2013); and multidisciplinary UK guidance for health professionals (2014).^15^ The Dutch and German multidisciplinary guidelines and the Danish GP guideline make use of the Grading of Recommendations Assessment, Development and Evaluation (GRADE) method to link levels of evidence to the recommendations included in the guidelines.^16^ The Dutch GP guideline and the multidisciplinary UK guidance for health professionals do not report levels of evidence in line with GRADE recommendations. According to the guidelines the following elements are important for the treatment of patients with MUS.

#### Doctor–patient relationship

All guidelines mentioned above specifically pay attention to the doctor–patient relationship and incorporate specific sections on this issue. There is high-quality evidence that patients with MUS present difficulties in the general practice encounter and challenge the GP–patient relationship;^13,14^ they frequently evoke feelings of frustration and irritation in GPs who experience difficulties in explaining MUS to patients. The Dutch multidisciplinary guideline states that a good doctor–patient relationship is associated with patient satisfaction and improved health outcomes, and is an important condition for a good treatment course.^11^ All guidelines recommend the strengthening of the doctor–patient relationship, which can be achieved by doctors recognising the patient’s illness, taking the patient and their symptoms seriously, and demonstrating empathy.

#### Doctor–patient communication

All guidelines emphasise the importance of doctor–patient communication. There is high-quality evidence that good doctor–patient communication in the consultation is essential for the treatment of MUS as patients seek a shared understanding of their symptoms.^12^ Guidelines recommend using standard approaches to exploring symptoms, such as the ideas, concerns, and expectations model, employing open questions. Consultations should aim to validate the patient’s distress and provide a detailed insight into the patient's biopsychosocial background, needed for a shared understanding of the symptoms. Based on lower levels of evidence, the Dutch GP guideline and the German and UK guidelines specifically pay attention to the provision of a summary by the GP as a communication tool.^12,13^ Such a summary should include the topics that have been discussed in the consultation. It gives the patient the opportunity to check whether the GP understands the problem and to add their perspective to it. The guidelines also state that explicit communication about expected results of biomedical investigations is essential.^11–15^ When discussing treatment, the Danish guideline states that the GP should communicate with the patient in an open and accommodating manner, in which the advantages and disadvantages of further testing and treatment can be discussed.^14^

#### Explanation of symptoms

Although this has not been tested specifically in randomised controlled trials (RCTs), all guidelines state that it is important to provide a targeted and tangible explanation in the patient’s language about the cause of their symptoms. Information obtained during the structured exploration of the symptoms should be incorporated into this explanation. The UK guideline is the most explicit about the value of providing explanations.^15^ It states that patients benefit from an 'explanation that makes sense, removes any blame from the patient, and generates ideas about how to manage the symptoms'. The Danish guideline suggests that patients perceive biological explanatory models as specifically useful.^14^

#### A stepped-care approach

All guidelines recommend a stepped-care approach.

The Dutch, Danish, and German guidelines describe three stages of MUS severity, which lack clear cut-off points. These stages of severity correspond with different management options using a stepped-care approach (Box 1).^11–14^ The German guideline focuses on the risk of progression to chronic MUS based on evidence from follow-up studies.^12^ Risk determinants for chronic MUS are: multiple symptoms; duration of symptoms; functional impairment; psychosocial stress; psychological comorbidity; and experienced difficulties in the doctor–patient relationship.^12^

**Box 1.** Stepped-care approach in clinical guidelines for MUS

Dutch GP guideline		Danish GP guideline		German multidisciplinary guideline		Dutch multidisciplinary guideline	
**Mild MUS**	Psycho-education (Self-)management advice Shared time-contingent plan Follow-up	**Symptoms and mild functional disorders**	Normalisation, explanation, biopsychosocial approach Follow-up	**Step 1**	General principles of therapy (empathy, watchful waiting, acknowledgement of the symptoms, explanation) Therapy by GP or medical specialist, or psychosomatic primary health care	**Mild MUS**	Biopsychosocial approach by GP Psycho-education Short-term CBT
**Moderate** **MUS**	Psychosomatic physio/exercise therapy Mental health nurse practitioner Social psychiatric nurse	**Moderate functional disorders**	Explanations and TERM model Regular consultations Cooperation with specialist (in charge of assessment, treatment plan, and supervision)	**Step 2**	Regular consultations Therapy by GP or medical specialist PLUS psychotherapy Pain as core symptom: antidepressant Pain not as core symptom: antidepressant in case of psychiatric comorbidity	**Moderate MUS**	Case management by medical specialist, psychiatrist or GP Medication (for comorbidity) CBT
**Severe** **MUS**	Multidisciplinary team / treatment centre	Severe functional disorders	Specialist clinic Multidisciplinary treatment CBT and GET Consider pharmacological treatment	**Step 3**	Specialist clinic with multidisciplinary treatment	**Severe MUS**	CBT Treatment by a multidisciplinary team in tertiary care

CBT = cognitive behavioural therapy. GET = graded exercise therapy. MUS = medically unexplained symptoms. TERM = The Extended Reatribution and Management model.

Based on good clinical practice, the Danish guideline recommends delivering proactive care and making regular follow-up appointments at fixed intervals during the course of treatment in complex cases.^14^ Furthermore, it suggests that it is important that one care provider, preferably the GP, keeps control and coordinates the care process. The Dutch GP guideline states that this could also be a social psychiatric nurse, psychologist, or occupational health physician.^13^

All guidelines state that the more severe or complex the symptoms and limitations, the more intense and complex the treatment needed for patient recovery. The most severely affected patients need a close collaboration between professionals with a divergent range of skills and expertise in secondary or tertiary care (that is, the final step in the stepped-care approach).

### Cochrane Reviews on MUS in primary care

Four recent Cochrane Reviews summarise the available evidence on the management of MUS in primary care. Van Dessel *et al* studied the efficacy of non-pharmacological (psychological and physical) interventions for MUS in adults ^17^while Kleinstauber *et al* assessed the efficacy of pharmacological interventions.^18^ Hoedeman *et al* assessed the effectiveness of consultation letters to support primary care physicians in their management of patients with MUS.^19^ Finally, Rosendal *et al* aimed to assess the clinical effectiveness of enhanced care interventions, delivered by professionals providing frontline primary care for adults with MUS.^20^ Recently, these reviews have been summarised as an illustration of the uncertainty in the management of MUS patients.^21^ In the sections that follow, we will describe the evidence from these Cochrane Reviews in a narrative way. We first describe evidence from efficacy trials involving specialised treatment, then evidence from effectiveness trials conducted in routine primary care.

#### Effects of psychological interventions

In a Cochrane Review from 2014 the efficacy of psychological treatments for MUS in adults was analysed.^17^ The meta-analysis comparing psychological therapy delivered in primary or secondary care (hospital and outpatient clinics) with usual care or placement on a waiting list found that the use of psychological therapy resulted in less severe symptoms at the end of the treatment. The Cochrane Review also demonstrated that, in the 14 studies analysed, cognitive behavioural therapy (CBT) delivered by specially-trained healthcare professionals in primary care or in specialised settings, is more effective than usual care. CBT reduces symptom severity, but effect sizes are small. However, effects seem to be sustained after 1 year of follow-up. Stratified analyses of other psychological treatment strategies (for example, problem solving therapy, behavioural therapy, mindfulness, or psychodynamic therapies) did not show significant effects based on a small number of studies. We conclude that psychological treatments, more specifically CBT, provided by specially-trained professionals reduce symptom severity in patients with MUS, but effect sizes are small.

#### Effects of physical interventions

Although many GPs are inclined to refer patients with MUS for active therapies such as walking, running, and exercising, the evidence of their efficacy is absent. The authors of a 2014 Cochrane Review on non-pharmacological interventions found no trials on the efficacy of physical therapies, such as running therapy, graded exercise or activation therapy, for patients with MUS.^17^

#### Effects of pharmacological interventions

A Cochrane Review from 2014 provides an overview of the current status of research on the efficacy and acceptability of pharmacological treatments for patients with MUS.^18^ The meta-analysis is based on 26 RCTs involving 2159 participants aged 18–64 years presenting with long-lasting MUS and significant impairment of functioning. The follow-up period in the studies ranged between 2 and 12 weeks. The main results from this meta-analysis showed that:

1. there is no difference in efficacy between tricyclic antidepressants (TCAs) and placebo on the severity of symptoms; 
2. new-generation antidepressants such as selective serotonin reuptake inhibitors (SSRIs) and serotonin and norepinephrine reuptake inhibitors (SNRIs) may have a moderate effect on symptom severity versus placebo; and
3. natural products, for example herbal remedies such as St John’s wort, may reduce the severity of symptoms and other secondary outcomes compared with placebo.

Comparisons in this meta-analysis are based on a limited number of studies with small numbers of participants. Furthermore, participants were mainly recruited outside of primary care. The methodological quality of included studies is low and the risk of bias high. Therefore, we conclude that there is little evidence for the efficacy of medication for MUS in primary care.

#### Effects of consultation letters

A Cochrane Review from 2010 on the effectiveness of consultation letters to support primary care physicians in their treatment of patients with MUS analysed six RCTs involving 449 patients.^19^ The authors reported limited evidence for improved physical functioning, but lower medical costs for an intervention consisting of screening by a psychiatrist followed by a letter providing the GP with advice on patient treatment. Joint consultation by a psychiatrist and GP, followed by a letter with advice for the GP, resulted in a slight reduction in the severity of the symptoms. However, all studies were performed in the US, study populations were small, and the studies were of moderate quality.

#### Effects of enhanced primary care

Enhanced care entails treatment models applied within the primary care setting by frontline primary care professionals instead of by trained therapists. It includes techniques of explanation, reattribution and some components of CBT. A Cochrane Review from 2013 reported on the outcomes from six effectiveness studies^20^ and found that treatments varied from very brief interventions delivered within a normal consultation to structured programmes of longer duration involving several appointments. Findings of the trials were mixed. Trials of brief reattribution and related interventions within ordinary consultations did not show a benefit to patient outcomes, although the training GPs received within the studies improved their communication skills, professional attitudes and confidence when dealing with patients with MUS. Trials of more intensive interventions with repeated consultations suggested clinically meaningful patient benefit, but only included small numbers of patients; such interventions need to be tested in larger trials.^20^

## Discussion

### Summary

The available Cochrane Reviews provide some guidance for the management of patients with MUS in primary care. The following were found with regard to patient outcomes: 

1. the effectiveness of enhanced care by primary care professionals is currently limited;
2. the efficacy of psychological treatment provided by specialists is well documented and CBT is the psychological treatment with the strongest current evidence; 
3. the role of pharmacotherapy is very limited; 
4. evidence for physical treatment is absent; and 
5. the effectiveness of consultation letters is limited.

Although largely based on consensus, the guidelines provide GPs with greater insight. The following specific communication skills are recommended: systematically exploring patient’s ideas, concerns, and expectations, and providing a targeted and tangible explanation for their symptoms. If patients have mild-to-moderate MUS, GPs should provide them with practical and constructive advice that is easily applicable to their daily lives, but the guidelines provide limited guidance on how to achieve this. Finally, guidelines advocate a stepped-care approach for all patients with MUS in which the most severely affected patients benefit from a multidisciplinary approach. The severity of the symptoms and the functional limitations guide the management options in the stepped-care model.

### Strengths and limitations

It is important to notice that we did not include evidence from specific syndromes such as irritable bowel syndrome or chronic fatigue syndrome in this narrative review. A number of functional syndromes have been researched and evidence from this area may further contribute to our current knowledge about MUS in general.

Systematic reviews, and consequently guidelines based largely on such reviews, are most appropriate to address relatively simple questions about the effect of interventions on clearly defined conditions. Systematic reviews and RCTs may be less appropriate in patients with MUS where boundaries are ambiguous and patients heterogeneous. Other research methods are necessary to generate new and important knowledge in this field.^22^ Moreover, as experienced practitioners, many GPs have developed successful strategies for patients with MUS that others may learn from.^23^

While CBT delivered by specialists has been shown to be effective, application of CBT by primary care professionals shows less effect. Regarding various implementations of reattribution techniques, evidence for patient benefit when applied by primary care professionals in routine care is lacking, but the techniques have not been tested in efficacy trials. An exploration of which techniques are feasible to apply in routine care and how to integrate them in primary care deserves more attention in future research.

### Implications for clinical practice

The clinical guidelines do provide some guidance, but their evidence base is limited and several barriers may prevent the application of the recommendations.

For example, GPs consider explanation and reassurance important in patients with MUS, but may experience many difficulties in explaining symptoms constructively^15^ and providing patients with specific advice and self-management strategies.^24^ As training in enhanced primary care techniques leads to changes in GP attitudes and awareness of MUS,^25^ work is needed to translate the positive effects on the GPs into better patient outcomes.

In order to overcome the clinical challenges more evidence is needed on: 

1. the effects of strengthening the doctor–patient relationship on the course and prognosis of MUS; 
2. the influence of specific consultation skills (for example, systematical exploration of patient ideas, concerns, and expectations, providing a summary and a personalised explanation) in consultations about MUS; 
3. the effects of physical therapy for patients with MUS; and
4. ways to deliver psychological treatment more effectively in primary care.

In conclusion, current systematic reviews indicate that psychological treatment delivered by specialists, especially CBT, has the strongest evidence for patient benefit. Training GPs to deliver brief reattribution type interventions in routine consultations improves their skills and attitudes but there is no evidence of improved patient outcomes. Guidelines concur that doctor–patient communication is key; they emphasise the importance of exploring patients’ ideas, concerns, and expectations, providing acceptable explanations and offering advice on symptom management. Severe and complex cases should be managed in collaboration with specialists in a stepped-care approach.
